# Marine Carotenoids against Oxidative Stress: Effects on Human Health

**DOI:** 10.3390/md13106226

**Published:** 2015-09-30

**Authors:** Maria Alessandra Gammone, Graziano Riccioni, Nicolantonio D’Orazio

**Affiliations:** 1 Human and Clinical Nutrition Unit, Department of Medical Oral and Biotechnological Sciences, Via Dei Vestini, “G. D’Annunzio” University, Chieti 66013, Italy; E-Mails: griccioni@hotmail.com (G.R.); ndorazio@unich.it (N.D.); 2 Cardiology Unit, San Camillo De Lellis Hospital, Manfredonia 71043, Foggia, Italy

**Keywords:** marine carotenoids, oxidative stress, antioxidants, inflammatory diseases

## Abstract

Carotenoids are lipid-soluble pigments that are produced in some plants, algae, fungi, and bacterial species, which accounts for their orange and yellow hues. Carotenoids are powerful antioxidants thanks to their ability to quench singlet oxygen, to be oxidized, to be isomerized, and to scavenge free radicals, which plays a crucial role in the etiology of several diseases. Unusual marine environments are associated with a great chemical diversity, resulting in novel bioactive molecules. Thus, marine organisms may represent an important source of novel biologically active substances for the development of therapeutics. In this respect, various novel marine carotenoids have recently been isolated from marine organisms and displayed several utilizations as nutraceuticals and pharmaceuticals. Marine carotenoids (astaxanthin, fucoxanthin, β-carotene, lutein but also the rare siphonaxanthin, sioxanthin, and myxol) have recently shown antioxidant properties in reducing oxidative stress markers. This review aims to describe the role of marine carotenoids against oxidative stress and their potential applications in preventing and treating inflammatory diseases.

## 1. Introduction

Oceans cover most of the earth’s surface, constituting a wide resource for the discovery of potential therapeutics. Over the last decades, numerous substances with interesting pharmaceutical activities have been identified in marine organisms. The diversity of marine environments provides an important source of bioactive compounds. This may lead to potential new drug candidates of natural origin with efficacy and low toxicity in the therapeutic strategy against many diseases characterized by cellular redox state alterations.

In recent years it has become evident that the oxidation of lipids is a fundamental step in the pathogenesis of several diseases, in both adult and infant patients. Lipid peroxidation is a process naturally generated in small amounts in the body, mainly by the effect of some reactive oxygen species (ROS), such as hydroxyl radical and hydrogen peroxide. Both enzymatic and non-enzymatic natural antioxidant defenses exist; however, these protective mechanisms may be overcome. Then a self-propagating chain-reaction starts and oxidative stress can result in significant tissue damage.

Oxidative stress and chronic inflammation are the main pathophysiological factors contributing to the development of chronic inflammatory diseases, such as diabetes, atherosclerosis, and hypertension. Appropriate and effective interventions, including nutrition, pharmacology, and physical exercise, are necessary in order to activate the expression of cellular antioxidant systems, thus preventing inflammatory and degenerative processes. In fact, inflammatory diseases derive from a continuum of patho-physiological processes: for example, cardiovascular disorders advance from a local redox disequilibrium to endothelial dysfunction, inflammation, and excessive vascular remodeling, which slowly leads to atherosclerosis and subsequent cardiovascular accidents such as coronary artery disease, myocardial infarction, and stroke [1]. A nutritional approach through natural antioxidant substances represents an important new frontier in both the prevention and treatment of cardiovascular diseases. Scientific evidence supports the beneficial roles of phytochemicals against some inflammatory and chronic diseases. Several naturally-occurring antioxidant bioactives have been associated with their prevention.

For example, many carotenoids with great antioxidant properties displayed a risk reduction both in epidemiological studies and supplementation human trials, indicating the presence of a strong link between oxidative stress, a pro-inflammatory systemic environment, and a wide number of chronic diseases [2]. Consequently, consistent dietary improvement may shift human health toward decreased morbidity and mortality as well as a better quality of life.

## 2. Oxidative Stress: The Role of Antioxidants

**Figure 1:**
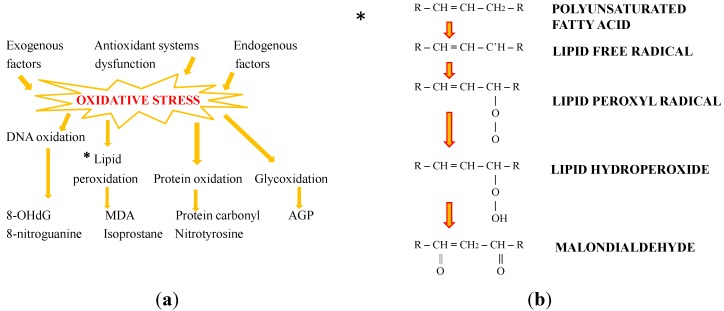
Sources, effects, and markers of oxidative stress. In detail, (**a**) schematic steps of MDA formation from polyunsaturated fatty acids (MDA: malondialdehyde; AGP: advanced glycation end-products; 8-OHdG: 8-hydroxy-2′-deoxyguanosine); (**b**) Lipid peroxidation and MDA production.

ROS are molecules containing oxygen, with unpaired valence electrons. They are generated as a natural product of normal cellular functioning and oxygen metabolism and have important roles in both cell signaling and intercellular homeostasis. ROS effects on cells include not only roles in apoptosis (programmed cell death) but also positive effects, such as the induction of host defense genes [3], the stimulation of the adaptive immune system via the recruitment of leukocytes, and mobilization of ionic transport systems in the so-called redox or oxidative signaling. However, ROS levels can augment dramatically due to environmental stress, such as ionizing radiation, UV, or heat exposure [4]; this increase can result in significant cellular damage, which cumulatively constitutes oxidative stress. ROS can damage DNA, RNA, and proteins; they can determine oxidations of both amino acids in proteins and polyunsaturated fatty acids in lipids and can oxidatively inactivate specific enzymes by oxidation of co-factors, thus contributing to the physiology of aging. Oxidative stress caused by the imbalance between ROS and biological antioxidant systems and consequent oxidative stress can lead to modification of these macromolecules (Figure 1); subsequently, in the case of excessive amounts, ROS can determine deleterious effects [5]. Free radicals play a crucial role in the progression of many pathologies, such as atherosclerotic processes [6], myocardial and cerebral ischemia [7], renal failure [8], rheumatoid arthritis [9], inflammatory bowel disease, retinopathy of prematurity, asthma, Parkinson’s disease, kidney damage, preeclampsia [10], and more general inflammation, as well as all the chronic degenerative diseases. In fact, ROS can attack the polyunsaturated fatty acids in the cell membrane, initiating a self-propagating chain reaction: this peroxidative rupture of cellular membranes and the end-products of such lipoperoxidation reactions are dangerous for cells and tissues, in a self-propagating chain-reaction whereby the initial oxidation of only a few molecules can result in significant tissue damage. Oxidative stress is a powerful contributor to aging, although the accumulation of oxidative damage and its implications for senescence depends on the particular tissue type where it occurs. Normally, cells defend themselves against ROS damage through intracellular and extracellular defenses, in particular through enzymes such as superoxide dismutases (SOD), catalases (CAT), lactoperoxidases, and glutathione peroxidases. Exogenous antioxidants such as ascorbic acid (vitamin C), tocopherol (vitamin E), and polyphenols also play important roles in preventing ROS damage by scavenging free radicals.

In a similar manner, carotenoids’ pigments, which have been shown to possess strong antioxidant properties, have been attracting increasing attention due to their beneficial effects on human health, in particular because of their potential against cancer and cardiovascular diseases [11].

## 3. Bioactivity and the Protective Effects of Natural Carotenoids: New Perspectives from the Sea

Carotenoids are lipid-soluble pigments produced by some plants, algae, fungi, and bacterial species. They are responsible for some food’s orange-yellow hues. Carotenoids, which play a crucial role in the complex network of antioxidant phytochemicals, should certainly be constituents of a healthy diet. They are excellent light filters and efficient quenchers of both singlet oxygen and excited triplet state molecules. Their lipophilicity determines their peculiar sub-cellular distribution: they are more represented in membranes and lipophilic cell compartments. This makes them suitable photo-protectants, not only for plants but also for humans. In fact, carotenoids absorb light, thus providing photo-protection and defense from photo-oxidative damage not only to photo-synthetic organisms, but also to the eye and the skin. Skin protection involves the carotenes β-carotene and lycopene, while protection of the macula involves the xanthophylls zeaxanthin and lutein.

Carotenoids are antioxidants thanks to their ability to quench singlet oxygen, to be oxidized, and to be isomerized. The protection mechanisms involve singlet oxygen quenching and free radicals scavenging. However, they scavenge reactive free radicals and become carotenyl radicals after reaction through hydrogen abstraction: this process can lead to a switch from a beneficial antioxidant process to a damaging pro-oxidative one [12]. This potential antioxidant role has been suggested to be the main mechanism for their preventive effects against cancer and inflammatory diseases. About 700 carotenoids with different structures have been isolated from natural source; the evaluation of their pharmaceutical potential may be a promising field of medical research.

However, the carotenoid species so far studied for this purpose are restricted to a small number, including the dicyclic β-carotene, β-cryptoxanthin, canthaxanthin, α-carotene and lutein, and the acyclic carotenoid lycopene. Typical carotenoids, as well as marine ones, displayed a wide range of beneficial effects on human health.

In this respect, novel marine carotenoids, such as fucoxanthin, astaxanthin, zeaxanthin, and, more recently, rare marine carotenoids such as sioxanthin, saproxanthin, myoxol, and siphoxanthin, are gaining attention and need to be evaluated for their important potential as development materials for pharmaceuticals or functional foods, in order to prevent such disorders as cancer and cardiovascular diseases. Marine carotenoids are important bioactive compounds principally derived from algae, with antioxidant activities deriving from their chemical structure and interaction with biological membranes. These bioactive substances recently showed unique and remarkable properties that explain their potentially beneficial effects on human health.

The potential benefits of marine carotenoids have been studied particularly in astaxanthin and fucoxanthin, which are the main marine carotenoids [13]. Both carotenoids show strong antioxidant activity, which is attributed to quenching singlet oxygen and scavenging free radicals.

### 3.1. Astaxanthin

**Figure 2:**
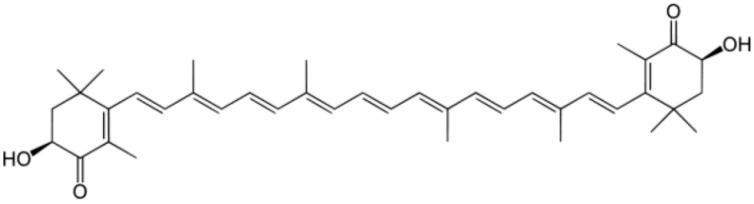
The molecular structure of astaxanthin.

Astaxanthin (Figure 2), a red carotenoid pigment belonging to the xanthophylls class, was shown to prevent lipid peroxidation in biological membranes and to support human health even more effectively than other antioxidants. It has been approved as a nutraceutical by the United States Food and Drug Administration since 1999 [14].

Astaxanthin, used in nutritional supplements, is usually a mixture of configurational isomers produced by the microalga *Haematococcus pluvialis*. As an antioxidant, it scavenges free radicals and other oxidants and protects the lipid bilayer from peroxidation with its polar ionic rings and non-polar conjugated carbon–carbon bonds, with an antioxidant property 10-fold greater than other carotenoids, including lutein, canthaxanthin, and β-carotene. In fact, astaxanthin and some derivatives can scavenge superoxide anion radicals (O_2_^−^) [15]; astaxanthin and its derivatives inhibit H_2_O_2_-mediated activation of the transcription factor NF-κB (nuclear factor kappa-light-chain-enhancer of activated B cells), which controls the expression of inducible genes such as heme oxygenase 1 (HO-1), which represents a marker of oxidative stress) and iNOS, implicated in the inflammatory response and engaged in cellular defenses against oxidative stress. Subsequently, astaxanthin also blocks its downstream cytokine production by modulating protein tyrosine phosphatase-1 expression [16]. Astaxanthin can interact with different radicals because the pattern of conjugated double bonds in its polyene backbone explains its ability to quench singlet oxygen [17] through the transfer of excitation energy to the carotenoid. In this process of quenching, astaxanthin remains intact so that it can undergo further cycles of singlet oxygen quenching [18]. As a consequence, astaxanthin supplementation not only lowers ROS levels but also leads to an important functional recovery of the antioxidant network including both the enzyme SOD, which alternately catalyzes the dismutation of O_2_^−^ into molecular oxygen (O_2_) and H_2_O_2_, and CAT, which protects the cell from oxidative damage by catalyzing the decomposition of H_2_O_2_^−^ to water and O_2_ [19]. Astaxanthin displayed antioxidant properties even stronger than vitamin E and β-carotene [20]: it provides protection against UV light damage and age-related diseases; it promotes the immune response in the liver and kidneys, but also in the eyes and joints; it protects phospholipids from peroxidation [21]; and it was responsible for important shifts in phlogistic response. For example, the treatment of *Helicobacter pylori*-infected mice with astaxanthin was demonstrated to reduce gastric inflammation and bacterial load and even to modulate cytokine release by splenocytes [22]. In addition, recent clinical studies showed a significant reduction in the cardiovascular risk markers of oxidative stress and inflammation [23], as well as an important improvement in blood status [24]. Astaxanthin has considerable potential for both the prevention and treatment of various chronic inflammatory disorders, such as cancer, asthma, rheumatoid arthritis, metabolic syndrome, diabetes, and diabetic nephropathy, as well as gastrointestinal, hepatic, and neurodegenerative diseases [2]. In addition, the effects of astaxanthin and *n*-3 polyunsaturated fatty acids in combination are synergistic: astaxanthin offered both antioxidant and anti-apoptotic activities to neutrophils, through improving their glutathione-based redox equilibrium.

Thus, habitual consumption of marine products such as fish and microalgae, which are natural sources of both astaxanthin and *n*-3 polyunsaturated fatty acids (PUFAs), may be associated with a significant improvement in immune response and may lower risks for both vascular and infective diseases [25].

Astaxanthin also displayed a neuro-protective property due to its antioxidant activities: in this respect, its age-dependent and region-specific antioxidant action in the mouse brain was recently investigated. Treated animals were given 2 mg/kg/day astaxanthin for four weeks. The level of non-enzymatic oxidative marker malondialdehyde (MDA), nitric oxide (NO), advanced protein oxidation product, and glutathione (GSH), and the activity of enzymatic antioxidants SOD and CAT were determined from the isolated brain regions. Astaxanthin supplementation markedly decreased the level of MDA, NO, and advanced protein oxidation product in the cortex, striatum, hypothalamus, hippocampus, and cerebellum. Treatment with astaxanthin increased the activity of CAT and SOD enzymes as well as the level of GSH in the brain. This noticeable improvement in oxidative markers was more important in the young group compared to the aged one, thus resulting in an age-dependent antioxidant effect of astaxanthin [26]. A similar result was achieved in animal models of autism (which had been obtained through prenatal exposure to valproic acid), where astaxanthin displayed neuro-protective effects due to its antioxidant mechanism [27]. In fact, oxidative stress leads to rapid changes in the antioxidant system, such as the dropping of the cellular endogenous antioxidant GSH, and may result in cell damage and even cell death, which may be responsible for autistic disorders [28]. Behavioral tests were conducted wherein oxidative stress markers (such as lipid peroxidation, advanced protein oxidation product, NO, and GSH) and the activity of SOD and CAT were estimated to confirm the mouse model of autism and assess the effect of astaxanthin. An increased level of oxidative stress was found by determining these different oxidative stress markers and astaxanthin significantly reduced the oxidative stress in the brain and liver, thus improving the behavioral disorder [27]. Hence, prenatal exposure to valproic in pregnant mice leads to the development of autism-like features, while astaxanthin improves the impaired behavior presumably by its antioxidant activity.

In addition, a very recent study demonstrates that astaxanthin also protects steroidogenesis from oxidative stress in Leydig cells. In fact, H_2_O_2_ induces oxidative stress and influences protein kinase A (PKA), a family of enzymes whose activity is dependent on cellular levels of cyclic adenosine monophosphate (c-AMP), which has several cell functions including regulation of glycogen, sugar, and lipid metabolism. Oxidative stress attenuates the post-PKA pathway, thus resulting in suppressed expression of the mature form of steroidogenic acute regulatory protein (StAR), a transport protein that regulates cholesterol transfer. Astaxanthin prevents the downregulation of the mature form of the StAR protein and restores steroidogenesis in the Leydig cell through a reduction of ROS formation caused by H_2_O_2_ [29].

Some evidence also revealed a potential therapeutic value of astaxanthin in pulmonary fibrosis treatment through promotion of myofibroblast apoptosis. A very recent study investigated the anti-fibrotic effect of astaxanthin on the promotion of myofibroblast apoptosis based on dynamin-related protein-1 (Drp1)-mediated mitochondrial fission *in vivo* and *in vitro* [30]. Results showed that astaxanthin can inhibit lung parenchymal distortion and collagen deposition, as well as promote myofibroblast apoptosis. Astaxanthin demonstrated pro-apoptotic function in myofibroblasts by contributing to mitochondrial fission, thereby leading to apoptosis by increasing Drp1 expression and enhancing Drp1 translocation into the mitochondria. Drp1-associated genes, such as Bcl-2-associated X protein, cytochrome c, tumor suppressor gene p53, and a p53-upregulated modulator of apoptosis, were highly upregulated in the astaxanthin group [30]. Hence, astaxanthin provides a potential preventive and therapeutic strategy in pulmonary fibrosis by promoting myofibroblast apoptosis through a Drp1-dependent molecular pathway.

Therefore, daily consumption of such marine products is a beneficial strategy in human health management. It can also help with fighting oxidative stress in healthy subjects whose free radical production is accentuated because of physical exercise, such as athletes [31].

These health-promoting effects of astaxanthin make it a high-value carotenoid and a novel potential treatment for oxidative stress and inflammation, not only against cardiovascular pathology [32] but also against other important inflammatory diseases.

### 3.2. Fucoxanthin

**Figure 3:**
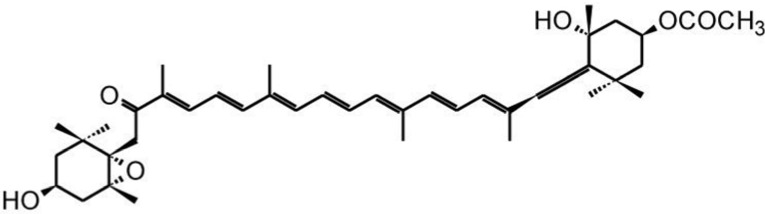
The molecular structure of fucoxanthin.

Fucoxanthin (Figure 3), another carotenoid, can be found in brown seaweeds such as *Undaria pinnatifida*, *Hijikia fusiformis*, *Laminaria japonica*, and *Sargassum fulvellum*. It belongs to the class of xanthophylls and non-provitamin A carotenoids. Its structure possesses an unusual allenic bond, an epoxide group, and a conjugated carbonyl group in a polyene backbone, which confers antioxidant properties [33]. Dietary ingested fucoxanthin is converted to amarouciaxanthin A, which is stored in abdominal white adipose tissue (WAT) via fucoxanthinol in mice. It is hydrolyzed to fucoxanthinol in the gastrointestinal tract by digestive enzymes such as lipase and cholesterol esterase, and then converted to amarouciaxanthin A in the liver [34]. These metabolites are considered to be the active forms exerting physiological and biological functions in the body. Fucoxanthin was demonstrated to be effective in the reduction of major cardiovascular risk factors such as obesity, diabetes, hypertension, chronic inflammation, plasma, and hepatic triglyceride levels, as well as cholesterol concentrations [35]. In particular, fucoxanthin was found to induce in white adipose tissue (WAT) both protein and mRNA expression of UCP1, a protein situated in the mitochondrial inner membrane dissipating the pH gradient of oxidative phosphorylation, thus releasing chemical energy as heat, and which is normally expressed only in brown adipose tissue (BAT). UCP1 induction by fucoxanthin metabolites accumulated in WAT increases the amount of energy released as heat in fat tissue and leads to oxidation of fatty acids and heat production in WAT [36]. Physiologic bodily metabolism determines heat production; this process is named thermogenesis, and UCP-1 dissipates the pH-gradient generated by oxidative phosphorylation, through releasing chemical energy as heat. UCP1 gene expression, which is stimulated by many factors, such as cold, β3-agonists, adrenergic stimulation, and thyroid hormones, represents a significant part of body energy expenditure, its dysfunction being an important cause of weight gain and a significant cofactor for the development of obesity. Fucoxanthin augments the amount of energy that is released as heat in fat tissue, thus stimulating thermogenesis [36]. UCP-1 and mRNA could be detected in WAT when experimental animals received *Undaria* lipids containing fucoxanthin: 0.2% fucoxanthin in their diet significantly attenuated weight gain in mice by increasing UCP-1 expression. This UCP1 induction in white adipose tissue (WAT) by fucoxanthin and its derivatives leads to fatty acid oxidation and heat production in WAT [35]. This adaptive thermogenesis plays a crucial role in energy expenditure as heat, in order to limit weight gain and favor weight loss.

Fucoxanthin was found to promote not only UCP1 protein and mRNA expression in WAT of obese animals but also β3-adrenergic receptor (Adrb3), which is responsible for lipolysis and thermogenesis [36]. This increased sensitivity to sympathetic nerve stimulation may lead to a further upregulation of fat oxidation in WAT. A clinical study on humans [37] tested the effects of 16-week supplementation with fucoxanthin in obese patients with non-alcoholic fatty liver disease (NAFLD), providing a significant reduction of body weight, fat, and systolic/diastolic blood pressure, decreased levels of TG, C-reactive protein (CRP), and some enzymes such as glutamic pyruvic transaminase (GPT), glutamic oxaloacetic transaminase (GOT), and gamma-glutamyl transpeptidase (gamma-GT), and a significant increase in resting energy expenditure (REE) measured by indirect calorimetry. Supplementation with 4.0 mg/day fucoxanthin led to an important increase in REE and an even greater increase was observed in the group taking fucoxanthin at a dose of 8 mg. A significant reduction in body weight and fat in obese subjects results in the downregulation of inflammatory markers and helps prevent metabolic syndrome. The potential anti-diabetic action of fucoxanthin is attributable to the ability of this marine bioactive to induce weight loss and WAT reduction, so that it helps decrease insulin resistance. In fact, the chronic low-grade inflammation elicited by pro-inflammatory mediators in the WAT leads to decreased insulin sensitivity [38]. A recent study showed that the metabolite fucoxanthinol also prevents inflammation and insulin resistance by inhibiting NO and prostaglandin E2 (PGE2) production through the downregulation of both iNOS and cyclooxygenase-2 (COX-2) mRNA expression, which are related to the pathogenesis of inflammation, as well as adipokine secretion in WAT [39].

In addition, fucoxanthin might alter the plasma leptin level in order to achieve its anti-obesity action. Many previous studies reported that leptin secretions are elevated due to the accumulation of fat in adipocytes; leptin could control body weight and adipose fat pads through the regulation of energy expenditure. In particular, Park *et al.* [39] performed a study evaluating the beneficial effect of *Undaria pinnatifida* ethanol extract on C57BL/6J mice and found that fucoxanthin could significantly decrease the plasma leptin level and that it was associated with a significant decrease in the epididymal adipose tissue weight. In this study, fucoxanthin supplementation reduced the adipocyte size remarkably compared to the control group. Fasting blood glucose, plasma leptin, and insulin levels were significantly higher in the control group by 1.5- to 2.3-fold. Fucoxanthin significantly lowered blood glucose levels by 19.8% and blood insulin levels by about 33%, compared to the control. The plasma leptin concentration showed a positive correlation with body weight and was lower after fucoxanthin supplementation. Another relevant study displayed that fucoxanthin downregulates stearoyl-coenzyme A desaturase-1 (SCD1), with subsequent improvement of insulin and leptin sensitivity, thus contributing to the prevention of obesity [36].

Another metabolic benefit of fucoxanthin was the promotion of docosahexaenoic acid (DHA) synthesis in the rodent liver, thus resulting in improved lipid profiles. Supplementation of fucoxanthin or its derivatives consistently attenuated body and visceral fat weight gain as well as lipid accumulation in the liver, decreases insulin resistance, and improves the plasma lipid profile in rodents fed a high-fat diet. However, it should be noted that in diabetic/obese KK-Ay mice with genetically compromised insulin signaling, fucoxanthin might increase the plasma levels of cholesterol and low-density lipoproteins. These beneficial metabolic effects of fucoxanthin are apparently mediated by the hormones leptin and adiponectin through their common target, adenosine monophosphate-activated protein kinase (AMK), resulting in a downregulation of lipogenic enzymes and an upregulation of lipolytic enzymes [40]. In addition, experiments on stroke-prone spontaneously hypertensive rats show the possible protective role of fucoxanthin against cerebrovascular accidents, even if the metabolic boost from taking fucoxanthin did not stimulate the central nervous system. Thus fucoxanthin might have a potential role in the modulation and prevention of human diseases, particularly in reducing the incidence of cardiovascular diseases [41]. Fucoxanthin was proved to be safe and without side effects, and provided numerous health benefits. Therefore, a fucoxanthin-rich diet could reduce body fat accumulation and modulate blood glucose and insulin levels through the regulation of cytokine secretions from WAT.

Apart from the metabolic benefits, fucoxanthin and its metabolite fucoxanthinol have recently been evaluated against proliferation of estrogen-sensitive MCF-7 and estrogen-resistant MDA-MB-231 breast cancer cell lines [42]. These cell lines were stimulated with 10–20 μM of fucoxanthin or fucoxanthinol, followed by cell viability assays and immunofluorescence to evaluate apoptosis, as well as mRNA and protein extractions for changes in NF-κB expressions and nuclear translocations. Fucoxanthin and fucoxanthinol reduced the viability of MCF-7 and MDA-MB-231 cells in a time-dependent manner as a result of increased apoptosis. In both cell lines, the modulatory action of fucoxanthinol on members of the NF-κB pathway was more pronounced than fucoxanthin in reducing nuclear levels of NF-κB members p65 and p52 and the transcriptional factor RelB [42]. Hence, fucoxanthinol and fucoxanthin could be effective for the treatment and/or prevention of breast cancer, thus opening new frontiers in anticancer research.

### 3.3. Zeaxanthin

**Figure 4:**
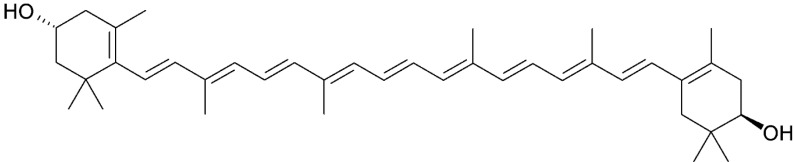
The molecular structure of zeaxanthin.

Zeaxanthin (Figure 4) is another oxygenated non-provitamin A carotenoid that consists of a 40-carbon hydroxylated compound identical to lutein [43]. Dietary sources of this xanthophyll include corn, eggs, orange, honeydew melon, and green leafy vegetables, but it can be also found in marine sources. *Gramella oceani* sp., a zeaxanthin-producing bacterium of the family Flavobacteriaceae, was recently isolated from marine sediment off coastal Taiwan [44]. Xanthophyll has also been identified in *Gramella planctonica* sp. nov., *Aquibacter zeaxanthinifaciens* sp. nov., and *Kordia aquimaris*, and in algae such as *Rhodophyta* spp. and *Spirulina* spp. [45].

The human retinal area involved in central vision is named macula lutea due to its yellow coloration from lutein, but it also contains zeaxanthin, whose localization is more centralized than lutein. Both lutein and zeaxanthin intake are positively correlated with augmented macular pigment density, which lowers the risk for age-related macular degeneration (AMD). Numerous population studies indicate lower rates of AMD among subjects with higher blood levels of zeaxanthin, mostly because of its antioxidant protection of retinal tissue but also because of its ability to filter out the damaging blue light [46]. Zeaxanthin not only directly scavenges ROS but also prevents protein, lipid, or DNA oxidative damage by regulating other antioxidant mechanisms such as intracellular GSH. This protective effect of zeaxanthin is comparable to α-tocopherol: supplementation with either zeaxanthin or α-tocopherol reduces oxidized glutathione (GSSG) and augments intracellular reduced glutathione levels and the GSH:GSSG ratio in response to oxidative stress [47]. In this sense, zeaxanthin acts as a direct antioxidant but also an indirect one, by regulating GSH synthesis and levels, so that the intracellular redox status upon oxidative stress is ameliorated while susceptibility to (H_2_O_2_)-induced cell death declines [48].

An important clinical trial demonstrating the long-term benefit of supplemental carotenoids was the Age-Related Eye Disease Study (AREDS), which meant to learn more about the natural history and risk factors of AMD and cataracts. It evaluated the effect of high doses of vitamin C, vitamin E, β-carotene, and zinc, showing that high levels of these antioxidants significantly decreased AMD progression and vision loss risk [49]. Subsequently, ARDS2 introduced some modifications to the initial formulation by substituting lutein and zeaxanthin for β-carotene, since prior studies had shown an increased risk of lung cancer in smokers. Lutein and zeaxanthin together appeared to be a safe and effective alternative to β-carotene.

Recent studies have shown that, in addition to traditional mechanisms, zeaxanthin can influence cell viability and function through various signal pathways or transcription factors.

It has been reported that zeaxanthin decreased the upregulation of vascular endothelial growth factor (VEGF) in the retina of diabetic rats and in apolipoprotein-deficient mice [50]. 

Zeaxanthin was very recently shown to block hypoxia-induced VEGF secretion in cultured human retinal pigment epithelial cells; additionally, it may have a broader effect on the control of angiogenesis caused by factors other than VEGF through inhibition of hypoxia-induced accumulation of hypoxia-inducible factors-1α (HIF-1α). Taken orally, zeaxanthin could be used as an adjunct to intravitreal anti-VEGF therapy, thus enabling a decreased frequency of injections with a consequently reduced risk of local side effects [51]. Therefore, it could be a promising agent to be explored for the prevention and treatment of a variety of retinal diseases associated with revascularization.

In addition, similarly to β-carotene, zeaxanthin was inversely correlated with common carotid artery stiffness, as well as elastic modulus and pulse wave velocity. The Beijing Atherosclerosis Study and the Los Angeles Atherosclerosis Study had already shown the inverse association between plasma lutein and early atherosclerosis; their follow-up trials and further studies confirmed that higher plasmatic levels of zeaxanthin could be protective against early atherosclerosis, too [52]. These results indicated that zeaxanthin may be beneficial not only to eyes but also to cardiovascular health.

Even if evidence from AREDS2 and other studies suggests that lutein and zeaxanthin could be more appropriate than β-carotene in supplements [53], more prolonged follow-up will provide further information on the biological mechanisms, duration of trial effects, and potential late effects of intervention with these antioxidants.

### 3.4. β-Cryptoxanthin

**Figure 5:**
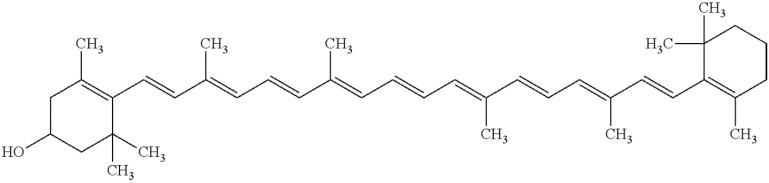
The molecular structure of β-cryptoxanthin.

β-Cryptoxanthin (Figure 5) is a xanthophyll with pro-vitamin A activity; its best dietary sources are orange fruits such as oranges, peaches, tangerines, and tropical fruits, especially papaya, but also marine sources, such as *Nanochlorum eucaryotum*, a novel marine alga with unusual biological characteristics [54].

Epidemiological studies showed that β-cryptoxanthin improves respiratory function and lowers lung cancer rates; prospective studies recognized its dietary intake as protective [55] since its antioxidant potential protects against the oxidative damage that can result in inflammation. In tissue culture, β-cryptoxanthin demonstrated a direct stimulatory effect on bone formation and, at the same time, an inhibitory effect on bone re-absorption [56]. Large prospective population-based studies, such as the European Prospective Investigation of Cancer Incidence (EPIC)-Norfolk study [57] as well as the Iowa Women’s Health Study [58], showed that increased β-cryptoxanthin intake was associated with a reduced risk of developing inflammatory diseases such as inflammatory poly-arthritis, synovitis, and rheumatoid arthritis. These studies did not show the same beneficial effect for β-carotene, lutein, and zeaxanthin; the influence of β-cryptoxanthin on some inflammatory markers is probably stronger than other carotenoids. Further epidemiologic studies showed that CRP and oxidized low-density lipoprotein (LDL)-cholesterol plasmatic levels are inversely linked to circulating antioxidants, including β-cryptoxanthin concentrations [59]. In addition, a recent report showed an inverse correlation between β-cryptoxanthin serum concentration and obesity, which was directly related to CRP in the general population instead [60]. Therefore, β-cryptoxanthin may also be associated with a decreased cardiovascular risk and consequently with a reduced morbidity and mortality.

In addition, the cancer preventive effect of β-cryptoxanthin has been widely described in population studies. The relation of head and neck cancer (HNC) risk with the intake of carotenoids was recently explored: the analysis included over 6000 subjects with oral, laryngeal, and pharyngeal cancer, categorized by quintiles of carotenoid intake from natural sources. Higher intake of β-cryptoxanthin was associated with a reduction of at least 18% in the rate of oral and pharyngeal cancer and a 17% reduction in the rate of laryngeal cancer. The overall protective effect of β-cryptoxanthin on HNC was stronger for subjects reporting greater tobacco or alcohol consumption [61]. Hence, a diet rich in carotenoids may protect against HNC, especially in persons with high risk.

A very recent human intervention study [62] focused on the therapeutic potential of β-cryptoxanthin individually and in combination with oxaliplatin in colon cancer: β-cryptoxanthin decreased the proliferation of cancer cells and cooperated with oxaliplatin to induce apoptosis through the negative regulation of NH_2_-terminally truncated p73 (ΔNP73). The administration of anti-tumoral drugs such as oxaliplatin can decrease in the presence of β-cryptoxanthin to achieve same percentage of growth inhibition. Thus, a putative novel therapeutic strategy for the treatment of colon cancer could be based on the combination of β-cryptoxanthin and oxaliplatin. The combined regimen produced greater benefits than either individual modality, without increasing side effects. Additionally, the concentration-limiting toxicity of oxaliplatin is reduced in presence of this antioxidant carotenoid [62].

In addition, the anti-metastatic effect of β-cryptoxanthin (0.2 μM) was assessed in Taiwanese and American populations using human hepatocarcinoma SK-Hep-1 cells [63]. Results revealed an additive inhibition on invasion, migration, and adhesion at 48 h of incubation. The anti-metastatic action of β-cryptoxanthin and multicarotenoids involved additive reduction on the activities of matrix metalloproteinase (MMP-2 and MMP-9) and the protein expression of Rho and Rac 1, but additive promotion on the protein expression of MMP tissue inhibitors (TIMP-1 and TIMP-2) [63]. However, more *in vivo* studies are needed to confirm these findings.

### 3.5. Rare Marine Carotenoids: Siphonaxanthin, Saproxanthin, and Myxol

**Figure 6:**
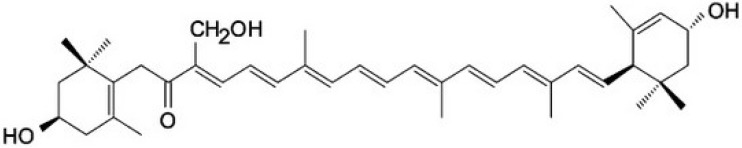
The molecular structure of siphonaxanthin.

Recent years have seen a rising trend in exploring microalgae as the demand for lutein and other carotenoids in global markets increased dramatically. New marine resources are now under examination and novel entities are emerging. Among these, siphonaxanthin (Figure 6) is a specific keto-carotenoid, present in edible green algae such as *Codium fragile*, *Caulerpa lentillifera*, *and Umbraulva japonica*, constituting approximately 0.1% of their dry weight, whose bio-functional properties are going to be identified [64]. Differently from fucoxanthin, siphonaxanthin does not possess either epoxide or an allenic bond in its structure, but contains an additional hydroxyl group on the 19th carbon that could contribute to its strong apoptosis-inducing effect. Siphonaxanthin seems to facilitate an efficient energy transfer of carotenoids to chlorophylls [65] and to have a light-harvesting function in underwater habitats [66].

Siphonaxanthin also proved to be a powerful inhibitor of human leukemia HL-60 cells’ viability through induction of their apoptosis (even more than fucoxanthin) due to its double cellular uptake. This strong pro-apoptotic effect was accompanied by reduced Bcl-2expression and subsequent activation of caspase-3 and upregulation of death receptor 5 (DR5) expression [67]. Tumor Necrosis Factor (TNF)-related apoptosis-inducing ligand (TRAIL) determines selective apoptosis in cancer cells by binding to the trans-membrane receptors TRAIL-R1/DR4 and TRAIL-R2/DR5 with no effects on normal cells [68]. This pathway constitutes an attractive strategy in anti-cancer research and in this sense siphonaxanthin may be a more potent growth inhibitor in cancer cells, compared to fucoxanthin, and could be a potential chemo-preventive or chemotherapeutic agent. It is already known that dietary carotenoids exert an anti-inflammatory effect by suppressing mast cell degranulation *in vivo*: astaxanthin, fucoxanthin, β-carotene, and zeaxanthin significantly block antigen-induced degranulation of rat basophilic leukemia cells (RBL-2H3) and bone marrow-derived mast cells through inhibition of antigen-induced translocation of the high-affinity IgE receptor FcεRI to lipid rafts [69]. In a similar way, siphonaxanthin was reported to exert inhibitory effects on the antigen-induced degranulation of mast cells, because it modifies the functions of lipid rafts by localizing in the cell membrane and inhibiting the translocation of FcεRI to lipid rafts [70].

In studies on human umbilical vein endothelial cells and the rat aortic ring, siphonaxanthin also showed a significant anti-angiogenic activity, due to signal transduction downregulation by fibroblast growth factor receptor-1 (FGFR-1) in vascular endothelial cells; in particular, siphonaxanthin suppresses the mRNA expression of fibroblast growth factor 2 (FGF-2), its receptor FGFR-1, and their *trans*-activation factor (EGR-1) [71]. This potential prevention of angiogenesis under pathological conditions, such as cancer, atherosclerosis, diabetic retinopathy, and rheumatoid arthritis [72], results in a promising approach in the prevention of cancer and other inflammatory, pro-angiogenic diseases.

**Figure 7:**
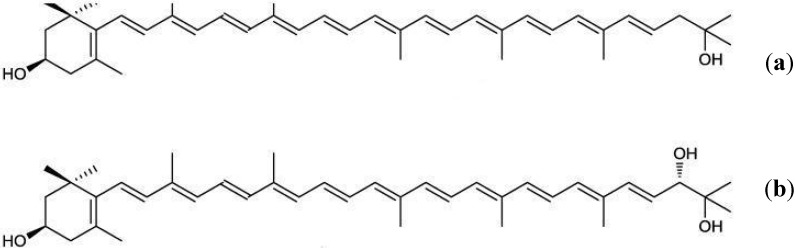
The molecular structures of saproxanthin (**a**) and myxol (**b**).

Recently, three novel marine bacteria belonging to the family Flavobacteriaceae have been isolated [73]. Two rare carotenoids, saproxanthin and myxol (Figure 7), were identified from these strains and reported to possess powerful antioxidant action. 3*R*-saproxanthin has been previously extracted from *Saprospira grandis* of the family Saprospiraceae; 3*R*,2′*S*-myxol has been found in marine bacterial strain P99-3, belonging to the family Flavobacteriaceae, and in cyanobacterium *Anabaena variabilis* [74]. If 2′-hydroxylase works on saproxanthin, this carotenoid is capable of being converted into myxol. The antioxidant potential of saproxanthin and myxol has been explained by their inhibitory activity against lipid peroxidation induced by free radicals in a rat brain homogenate and by their neuro-protective effect against l-glutamate toxicity on the neuronal hybridoma cell line [75].

Saproxanthin or myxol supplementation may determine reinforcement and stabilization of biological membranes, which decreases their permeability to oxygen and enhances protection against radical-induced peroxidation. The antioxidant activities of saproxanthin and myxol were even superior to those of zeaxanthin and β-carotene [73]. These rare novel and monocyclic marine carotenoids containing γ-carotene skeleton need to be carefully evaluated for their potential as development materials for pharmaceuticals or functional foods, in order to prevent oxidative stress-related diseases such as cancer and cardiovascular pathologies.

## 4. Antioxidant and Pro-Oxidant Activities of Carotenoids

The ability of dietary carotenoids to act as antioxidants in biological systems is dependent upon a number of factors. Even if their structure, in particular the conjugated double bond system, gives rise to many fundamental properties of these molecules, it also affects how these molecules are incorporated into biological membranes. This, in turn, alters the way these molecules interact with ROS, so that their behavior may be different *in vivo*, compared to in solution. The effectiveness of carotenoids as antioxidants is also dependent upon their interaction with other co-antioxidants, such as vitamins E and C [76]. In addition, rather than acting as proper scavenging agents, some carotenoids and their sub-products/metabolites activate the Nrf2-system, which triggers antioxidant gene expression in particular cells and tissues. The transcription factor Nrf2 (nuclear factor erythroid-2-related factor 2) activates the transcription of over 500 genes in the human genome, most of which have cytoprotective functions. Nrf2 acts via transcription of these genes, in order to raise antioxidant responses, mitochondrial biogenesis, energy metabolism, detoxification of carbon-containing xenobiotics or toxic metals, and autophagy of toxic protein aggregates and dysfunctional organelles, but it also greatly lowers many inflammatory responses [77]. Therefore, a great number of chronic diseases characterized by oxidative stress, inflammation and impaired mitochondrial function can be treated and/or prevented by raising Nrf2, at least in animal models. Nrf2 has been proposed to produce both lifespan and health span extension, given the many diseases of aging characterized by oxidative stress, inflammation, and mitochondrial dysfunction. 

Health-promoting nutrients such as carotenoids, terpenoids and phenolic compounds [78] act at least in part by raising Nrf2. Other health-promoting Nrf2-raising factors include low level oxidative stress (hormesis), exercise, and caloric restriction. Raising Nrf2 has been found to prevent and treat a large number of chronic inflammatory diseases, including various cardiovascular, kidney or lung diseases, toxic liver damage, metabolic syndrome, sepsis, autoimmune disorders, inflammatory bowel disease, human immunodeficiency virus (HIV) infection, and epilepsy. So, the induction of Nrf2-antioxidant response elements (ARE)-mediated antioxidant enzymes by carotenoids provides a cellular defense against oxidative stress.

In addition, some carotenoids, in particular astaxanthin, in combination with low concentrations of docosahexaenoic acid (DHA) and eicosapentaenoic acid (EPA), which are important nutritional ingredients found in fish oil, showed a synergistic antioxidant effect in a HepG2-C8-ARE-luciferase cell line system. The three compounds alone and in combination elevated cellular GSH levels, increased the total antioxidant activity, and induced mRNA expression of Nrf2 and Nrf2 downstream target genes NQO1 (which encodes NAD(P)H dehydrogenase-quinone 1), HO-1 (which encodes for heme oxygenase 1, an enzyme that is induced in response to stress), and GSTM2 (which encodes for the enzyme glutathione *S*-transferase Mu 2), with synergistic antioxidant effects at low concentrations. The Nrf2/ARE pathway plays an important role in the antioxidative effects induced by astaxanthin, DHA, and EPA [79].

On the other hand, carotenoids may lose their effectiveness as antioxidants at high concentrations or at high partial pressures of oxygen [77]. Their benefits not only seem to disappear but they can also be converted into negative effects. In particular, a 9-*cis*-β-carotene-rich diet reduced mRNA levels of CYP7a, the rate-limiting enzyme of bile acid synthesis [80], and consequently decreased cholesterol absorption in the intestine. β-Carotene also decreased the expression of interleukin (IL)-1a, vascular cell adhesion molecule-1 (VCAM-1), E-selectin, and genes involved in cholesterol metabolism and excretion, such as ABCG1, ABCG5, and ABCG8; this suggests its potential to inhibit atherosclerosis progression and, more generally, the inflammatory process in humans [32]. β-Carotene was also involved in body fat store control [81]: in mature adipocytes, it is metabolized to arachidonic acid (RA), which decreases the expression of peroxisome proliferator-activated receptor (PPAR)-alpha and CCAAT/enhancer-binding protein, which are key lipogenic transcription factors, and reduces the lipid content of mature adipocytes. A diet rich in β-carotene and fat tends toward energy expenditure; otherwise, adipocytes store energy as fat. In fact, circulating β-carotene levels are inversely correlated with risk of obesity and type 2 diabetes [82]. However, when β-carotene is administered as a pharmacological supplement, it has harmful effects in some sub-populations, thus acting as a pro-oxidant under specific conditions. Synthetic all-*trans* β-carotene seems to increase the incidence of lung cancer and cardiovascular disease in smokers [83]: the Alpha-Tocopherol Beta-Carotene Cancer Prevention (ATBC) Study, a randomized, double-blind, placebo-controlled primary prevention trial, reported that male smokers who took beta-carotene had an 18% increased incidence of lung cancer and an 8% increased overall mortality. The adverse effects of β-carotene were even stronger in the presence of alcohol consumption [84]. These results, in conjunction with those from the Beta-Carotene and Retinol Efficacy Trial (CARET) Study [85], continue to support the recommendation that β-carotene supplementation should be avoided by smokers.

## 5. Conclusions

In conclusion, natural bioactives may be used for therapeutic purposes, in order to protect cells against oxidative conditions. In particular, rare carotenoids need to be evaluated for their potential as development materials for pharmaceuticals and/or functional foods. It is hoped that this review will promote exploration of marine carotenoids best utilized.

## References

[1] Gori T., Nzel T.M. (2011). Oxidative stress and endothelial dysfunction: Therapeutic implications. Ann. Med..

[2] D’Orazio N., Gammone M.A., Gemello E., DeGirolamo M., Cusenza S., Riccioni G. (2012). Marine bioactives: Pharmacological properties and potential applications against inflammatory diseases. Mar. Drugs.

[3] Rada B., Leto T.L. (2008). Oxidative innate immune defenses by Nox/Duox family NADPH oxidases. Contrib. Microbiol..

[4] Devasagayam T.P.A., Tilak J.C., Boloor K.K., Sane K.S., Ghaskadbi S.S., Lele R.D. (2004). Free Radicals and Antioxidants in Human Health: Current Status and Future Prospects. J. Assoc. Phys. India.

[5] Liu J., Head E., Gharib A.M., Yuan W., Ingersoll R.T., Hagen T.M., Cotman C.W., Ames B.N. (2002). Memory loss in old rats is associated with brain mitochondrial decay and RNA/DNA oxidation: Partial reversal by feeding acetyl-l-carnitine and/or *R*-alpha-lipoic acid. Proc. Natl. Acad. Sci. USA.

[6] Palinski W., Rosenfeld M.E., Yla H.S., Gurtner G.C., Socher S.S., Butler S.W., Carew T.E., Parthasarathy S., Steinberg D., Witztum J.L. (1989). Low density lipoprotein undergoes oxidative modification *in vivo*. Proc. Natl. Acad. Sci. USA.

[7] Traysman R.J., Kirsch J.R., Koehler R.C. (1991). Oxygen radical mechanisms of brain injury following ischemia and reperfusion. J. Appl. Physiol..

[8] Erdogan C., Unlucerci Y., Turkmen A., Kuru A., Cetin O., Bekpinar S. (2002). The evaluation of oxidative stress in patients with chronic renal failure. Clin. Chim. Acta.

[9] Bodamyali T., Kanczler J.M., Millar T.M., Stevens C.R., Blake D.R. (2004). Free radicals in rheumatoid arthritis: Mediators and modulators. Oxid. Stress Dis..

[10] Mylonas C., Kouretas D. (1999). Lipid peroxidation and tissue damage. In Vivo.

[11] VanDenBerg H., Faulks R., Granado H.F., Hirschberg J., Olmedilla B., Sandmann G., Stahl W., Southon S. (2000). The potential for the improvement of carotenoid levels in foods and the likely systemic effects. J. Sci. Food Agric..

[12] Riccioni G., Speranza L., Pesce M., Cusenza S., D’Orazio N. (2012). Novel phytonutrient contributors to antioxidant protection against cardiovascular disease. Nutrition.

[13] Miyashita K. (2009). Function of marine carotenoids. Forum Nutr..

[14] Guerin M., Huntley M.E., Olaizola M. (2003). *Haematococcus* astaxanthin: Applications for human health and nutrition. Trends Biotechnol..

[15] Böhm F., Edge R., Truscott T.G. (2012). Interactions of dietary carotenoids with singlet oxygen (1O_2_) and free radicals: Potential effects for human health. Acta Biochim. Pol..

[16] Speranza L., Pesce M., Patruno A., Franceschelli S., DeLutiis M.A. (2012). Astaxanthin treatment reduced oxidative induced pro-inflammatory cytokinessecretion in U937: SHP-1 as a novel biological target. Mar. Drugs.

[17] Stahl W., Sies H. (2003). Antioxidant activity of carotenoids. Mol. Asp. Med..

[18] Wang S.L., He L.J., He T.B., Han W., Wang Q. (2015). Effect of astaxanthin on oxidative stress of red blood cells and peroxidation damage of membrane. Zhongguo Shi Yan Xue Ye Xue Za Zhi.

[19] Franceschelli S., Pesce M., Ferrone A., DeLutiis M.A., Patruno A., Grilli A., Felaco M., Speranza L. (2014). Astaxanthin Treatment Confers Protection against Oxidative Stress in U937 Cells Stimulated with Lipopolysaccharide Reducing O_2_^−^ Production. PLoS ONE.

[20] Shimidzu N. (1996). Carotenoids as singlet oxygen quenchers in marine organisms. Fish. Sci..

[21] Naguib Y.M.A. (2000). Antioxidant acitivities of astaxanthin and related carotenoids. J. Agric. Food Chem..

[22] Bennedsen M., Wang X., Willén R., Wadström T., Andersen L.P. (1999). Treatment of *H. pylori* infected mice with antioxidant astaxanthin reduces gastric inflammation, bacterial load and modulates cytokine release by splenocytes. Immunol. Lett..

[23] Yuan J.P., Peng J., Yin K., Wang J.H. (2011). Potential health-promoting effects of astaxanthin: A high-value carotenoid mostly from microalgae. Mol. Nutr. Food Res..

[24] Pashkow F.J., Watumull D.G., Campbell C.L. (2008). Astaxanthin: A novel potential treatment for oxidative stress and inflammation in cardiovascular disease. Am. J. Cardiol..

[25] Lara J.J., Economou M., Wallace A.M., Rumley A., Lowe G., Slater C., Caslake M., Sattar N., Lean M.E. (2007). Benefits of salmon eating on traditional and novel vascular risk factors in young, non-obese healthy subjects. Atherosclerosis.

[26] Al-Amin M.M., Akhter S., Hasan A.T., Alam T., Nageeb Hasan S.M., Saifullah A.R., Shohel C. (2015). The antioxidant effect of astaxanthin is higher in young mice than aged: A region specific study on brain. Metab. Brain Dis..

[27] Al-Amin M.M., Rahman M.M., Khan F.R., Zaman F., Mahmud Reza H. (2015). Astaxanthin improves behavioral disorder and oxidative stress in prenatal valproic acid-induced mice model of autism. Behav. Brain Res..

[28] Sajdel-Sulkowska E.M., Xu M., Koibuchi N. (2009). Increase in cerebellar neurotrophin-3 and oxidative stress markers in autism. Cerebellum.

[29] Wang J.-Y., Lee Y.-J., Chou M.-C., Chang R., Chiu C.-H., Liang Y.-J., Wu L.-S. (2015). Astaxanthin Protects Steroidogenesis from Hydrogen Peroxide-Induced Oxidative Stress in Mouse Leydig Cells. Mar. Drugs.

[30] Zhang J., Xu P., Wang Y., Wang M., Li H., Lin S., Mao C., Wang B., Song X., Lv C. (2015). Astaxanthin prevents pulmonary fibrosis by promoting myofibroblast apoptosis dependent on Drp1-mediated mitochondrial fission. J. Cell. Mol. Med..

[31] Gammone M.A., Gemello E., Riccioni G., D’Orazio N. (2014). Marine bioactives and potential application in sports. Mar. Drugs.

[32] Gammone M.A., Riccioni G., D’Orazio N. (2015). Carotenoids: Potential allies of cardiovascular health?. Food Nutr. Res..

[33] Hu T., Liu D., Chen Y., Wu J., Wang S. (2010). Antioxidant activity of sulfated polysaccharide fractions extracted from *Undaria pinnatifida in vitro*. Int. J. Biol. Macromol..

[34] Sangeetha R.K., Bhaskar N., Divakar S., Baskaran V. (2010). Bioavailability and metabolism of fucoxanthin in rats: Structural characterization of metabolites by LC-MS (APCI). Mol. Cell. Biochem..

[35] D’Orazio N., Gemello E., Gammone M.A., DeGirolamo M., Ficoneri C., Riccioni G. (2012). Fucoxantin: A treasure from sea. Mar. Drugs.

[36] Gammone M.A., D’Orazio N. (2015). Anti-obesity activity of the marine carotenoid fucoxanthin. Mar. Drugs.

[37] Abidov M., Ramazanov Z., Seifulla R., Grachev S. (2010). The effects of Xanthigen in the weight management of obese premenopausal women with non-alcoholic fatty liver disease and normal liver fat. Diabetes Obes. Metab..

[38] Matsuzawa Y., Shimomura I., Kihara S., Funahashi T. (2003). Importance of adipokines in obesity-related diseases. Horm. Res..

[39] Park H.J., Lee M.K., Park Y.B., Shin Y.C., Choi M.S. (2010). Beneficial effects of *Undaria pinnatifida* ethanol extract on diet-induced-insulin resistance in C57BL/6J mice. Food Chem. Toxicol..

[40] Muradian K., Vaiserman A., Min K.J., Fraifeld V.E. (2015). Fucoxanthin and lipid metabolism: A minireview. Nutr. Metab. Cardiovasc. Dis..

[41] Ikeda K., Kitamura A., Machida H., Watanabe M., Negishi H., Hiraoka J., Nakano T. (2003). Effect of *Undaria pinnatifida* on the development of cerebrovascular diseases in stroke prone spontaneously hypertensive rats. Clin. Exp. Pharmacol. Physiol..

[42] Rwigemera A., Mamelona J., Martin L.J. (2015). Comparative effects between fucoxanthinol and its precursor fucoxanthin on viability and apoptosis of breast cancer cell lines MCF-7 and MDA-MB-231. Anticancer Res..

[43] Holden J.M., Eldridge A.L., Beecher G.R. (1998). Carotenoid content of US foods: An update of the database. J. Food Compos. Anal..

[44] Shahina M., Hameed A., Lin S.Y., Lee R.J., Lee M.R., Young C.C. (2014). *Gramella planctonica* sp. nov., a zeaxanthin-producing bacterium isolated from surface seawater, and emended descriptions of *Gramella aestuarii* and *Gramella echinicola*. Antonie Van Leeuwenhoek.

[45] Hameed A., Shahina M., Lin S.Y., Lai W.A., Hsu Y.H., Liu Y.C., Young C.C. (2014). *Aquibacter zeaxanthinifaciens gen.* nov., sp. nov., a zeaxanthin-producing bacterium of the family Flavobacteriaceae isolated from surface seawater, and emended descriptions of the genera *Aestuariibaculum* and *Gaetbulibacter*. Int. J. Syst. Evol. Microbiol..

[46] Mares-Perlman J.A., Millen A.E., Ficek T.L., Hankinson S.E. (2002). The body of evidence to support a protective role for lutein and zeaxanthin in delaying chronic disease. J. Nutr..

[47] Giblin F.J. (2000). Glutathione: A vital lens antioxidant. J. Ocul. Pharmacol. Ther..

[48] Gao S., Qin T., Liu Z., Caceres M.A., Ronchi C.F., Chen C.Y.O., Yeum K., Taylor A., Blumberg J.B., Liu Y. (2011). Lutein and zeaxanthin supplementation reduces—H_2_O_2_^−^ induced oxidative damage in human lens epithelial cells. Mol. Vis..

[49] Chew E.Y., Clemons T.E., Agrón E., Sperduto R.D., Sangiovanni J.P., Davis M.D., Ferris F.L. (2014). Age-Related Eye Disease Study Research Group. Ten-year follow-up of age-related macular degeneration in the age-related eye disease study: AREDS report No. 36. JAMA Ophthalmol..

[50] Tanaka T., Shnimizu M., Moriwaki H. (2012). Cancer chemoprevention by carotenoids. Molecules.

[51] Rosen R., Vagaggini T., Chen Y., Hu D.N. (2015). Zeaxanthin inhibits hypoxia-induced VEGF secretion by RPE cells through decreased protein levels of hypoxia-inducible factors-1α. Biomed Res. Int..

[52] Dwyer J.H., Paul-Labrador M.J., Fan J. (2004). Progression of carotid intima-media thickness and plasma antioxidants: The Los Angeles Atherosclerosis Study. Arterioscler. Thromb. Vasc. Biol..

[53] Musch D.C. (2014). Evidence for Including Lutein and Zeaxanthin in Oral Supplements for Age-Related Macular Degeneration. JAMA Ophthalmol..

[54] Geisert M., Rose T., Bauer W., Zahn R.K. (1987). Occurrence of carotenoids and sporopollenin in *Nanochlorum eucaryotum*, a novel marine alga with unusual characteristics. Biosystems.

[55] Tanumihardjo S.A., Yang Z. (2005). Carotenoids: Epidemiology of Health Effects. Encyclopedia of Human Nutrition.

[56] Johnson E.J. (2002). The role of carotenoids in human health. Nutr. Clin. Care.

[57] Pattison D.J., Symmons D.P.M., Lunt M., Welch A., Bingham S.A., Day N.E., Silman A.J. (2005). Dietary beta-cryptoxanthin and inflammatory polyarthritis: Results from a population-based prospective study. Am. J. Clin. Nutr..

[58] Cerhan J.R., Saag K.G., Merlino L.A., Mikuls T.R., Criswell L. (2003). Antioxidant micronutrients and risk of rheumatoid arthritis in a cohort of older women. Am. J. Epidemiol..

[59] Kritchevsky S.B., Bush A.J., Pahor M., Gross M.D. (2000). Serum carotenoids and markers of inflammation in non-smokers. Am. J. Epidemiol..

[60] Suzuki K., Ito Y., Ochiai J. (2003). Relation-ship between obesity and serum markers of oxidative stress and inflammation in Japanese. Asian Pac. J. Cancer Prev..

[61] Leoncini E., Edefonti V., Hashibe M., Parpinel M., Cadoni G., Ferraroni M., Serraino D., Matsuo K., Olshan A.F., Zevallos J.P. (2015). Carotenoid intake and head and neck cancer: A pooled analysis in the International Head and Neck Cancer Epidemiology Consortium. Eur. J. Epidemiol..

[62] San Millan C., Soldevilla B., Martín P., Gil-Calderon B., Compte M., Pérez-Sacristán B., Donoso E., Peña C., Romero J., Granado-Lorencio F. (2015). β-Cryptoxanthin synergistically enhances the antitumoral activity of oxaliplatin through δNP73 negative regulation in colon cancer. Clin. Cancer Res..

[63] Chen H.Y., Yang C.M., Chen J.Y., Yueh T.C., Hu M.L. (2015). Multicarotenoids at Physiological Levels Inhibit Metastasis in Human Hepatocarcinoma SK-Hep-1 Cells. Nutr. Cancer.

[64] Sugawara T., Ganesan P., Li Z., Manabe Y., Hirata H. (2014). Siphonaxanthin, a Green Algal Carotenoid, as a Novel Functional Compound. Mar. Drugs.

[65] Akimoto S., Yokono M., Higuchi M., Tomo T., Takaichi S., Murakami A., Mimuro M. (2008). Solvent effects on excitation relaxation dynamics of a keto-carotenoid, siphonaxanthin. Photochem. Photobiol. Sci..

[66] Wang W., Qin X., Sang M., Chen D., Wang K., Lin R., Lu C., Shen J., Kuang T. (2013). Spectral and functional studies on siphonaxanthin-type light-harvesting complex of photosystem II from *Bryopsis corticulans*. Photosynth. Res..

[67] Ganesan P., Noda K., Manabe Y., Ohkubo T., Tanaka Y., Maoka T., Sugawara T., Hirata T. (2011). Siphonaxanthin, a marine algal carotenoids from green algae, effectively induces apoptosis in human leukemia (HL-60) cells. Biochim. Biophys. Acta.

[68] Srivastava R.K. (2001). TRAIL/Apo-2L: Mechanisms and clinical applications in cancer. Neoplasia.

[69] Sakai S., Sugawara T., Matsubara K., Hirata T. (2009). Inhibitory effect of carotenoids on the degranulation of mast cells via suppression of antigen-induced aggregation of high affinity IgE receptors. J. Biol. Chem..

[70] Manabe Y., Hirata T., Sugawara T. (2014). Suppressive effects of carotenoids on the antigen-induced degranulation in RBL-2H3 rat basophilic leukemia cells. J. Oleo Sci..

[71] Ganesan P., Matsubara K., Sugawara T., Hirata T. (2013). Marine algal carotenoids inhibit angiogenesis by down-regulating FGF-2-mediated intracellular signals in vascular endothelial cells. Mol. Cell. Biochem..

[72] Virmani R., Kolodgie F.D., Burke A.P., Finn A.V., Gold H.K., Tulenko T.N., Wrenn S.P., Narula J. (2005). Atherosclerotic plaque progression and vulnerability to rupture: Angiogenesis as a source of intraplaque hemorrhage. Atheroscler. Thromb. Vasc. Biol..

[73] Shindo K., Kikuta K., Suzuki A., Katsuta A., Kasai H., Yasumoto-Hirose M., Matsuo Y., Takaichi S. (2007). Rare carotenoids, (3*R*)-saproxanthin and (3*R*,2′*S*)-myxol, isolated from novel marine bacteria (Flavobacteriaceae) and their antioxidative activities. Appl. Microbiol. Biotechnol..

[74] Takaichi S., Mochimaru M., Maoka T. (2006). Presence of free myxol and 4-hydroxymyxol and absence of myxol glycosides in *Anabaena variabilis* ATCC 29413, and proposal of biosynthetic pathway of carotenoids. Plant Cell Physiol..

[75] Shindo K., Kimura M., Iga M. (2004). Potent antioxidant activity of cacalol, a sesquiterpene contained in *Cacalia delphiniifolia* Sleb et Zucc. Biosci. Biotechnol. Biochem..

[76] Young A.J., Lowe G.M. (2001). Antioxidant and prooxidant properties of carotenoids. Arch. Biochem. Biophys..

[77] Pall M.L., Levine S. (2015). Nrf2, a master regulator of detoxification and also antioxidant, anti-inflammatory and other cytoprotective mechanisms, is raised by health promoting factors. Sheng Li Xue Bao.

[78] Saw C.L., Yang A.Y., Guo Y., Kong A.N. (2013). Astaxanthin and omega-3 fatty acids individually and in combination protect against oxidative stress via the Nrf2-ARE pathway. Food Chem. Toxicol..

[79] Riccioni G., Gammone M.A., Tettamanti G., Bergante S., Pulchinotta F., D’Orazio N. (2015). Resveratrol and anti-atherogenic effects. Int. J. Food Sci. Nutr..

[80] Hubacek J.A., Bobkova D. (2006). Role of cholesterol 7alpha-hydroxylase (CYP7A1) in nutrigenetics and pharmacogenetics of cholesterol lowering. Mol. Diagn. Ther..

[81] Lobo G.P., Amengual J., Li H.N.M., Golczak M., Bonet M.L., Palczewski K., VonLintig J. (2010). Beta-Carotene Decreases PPAR-alpha Activity and Reduces Lipid Storage Capacity of Adipocytes in a beta-Carotene Oxygenase 1-dependent Manner. J. Biol. Chem..

[82] Ramakrishna V., Jailkhani R. (2008). Oxidative stress in non insulin dependent diabetes mellitus (NIDDM) patients. Acta Diabetol..

[83] (1994). The effect of vitamin E and beta carotene on the incidence of lung cancer and other cancers in male smokers. N. Engl. J. Med..

[84] Virtamo J., Pietinen P., Huttunen J.K., Korhonen P., Malila N., Virtanen M.J., Albanes D., Taylor P.R., Albert P., ATBC Study Group (2003). Incidence of cancer and mortality following alpha-tocopherol and beta-carotene supplementation: A post-intervention follow-up. JAMA.

[85] Omenn G.S., Goodman G., Thornquist M., Grizzle J., Rosenstock L., Barnhart S., Balmes J., Cherniack M.G., Cullen M.R., Glass A. (1994). The beta-carotene and retinol efficacy trial (CARET) for chemoprevention of lung cancer in high risk populations: Smokers and asbestos-exposed workers. Cancer Res..

